# Molecular Characterization of A Novel Effector Expansin-like Protein from *Heterodera avenae* that Induces Cell Death in *Nicotiana benthamiana*

**DOI:** 10.1038/srep35677

**Published:** 2016-11-03

**Authors:** Jing Liu, Huan Peng, Jiangkuan Cui, Wenkun Huang, Lingan Kong, Jihong Liu Clarke, Heng Jian, Guo Liang Wang, Deliang Peng

**Affiliations:** 1 State Key Laboratory for Biology of Plant Diseases and Insect Pests, Institute of Plant Protection, Chinese Academy of Agricultural Sciences, Beijing 100193, China; 2 Key Laboratory of Plant Pathology of Ministry of Agriculture, College of Plant Protection, China Agricultural University, Beijing 100193, China; 3 Plant Health and Biotechnology Division, Norwegian Institute of Bioeconomy Research, Høgskoleveien 7, 1430 Ås, Norway; 4 Department of Plant Pathology, Ohio State University, Columbus, OH 43210, USA

## Abstract

Cereal cyst nematodes are sedentary biotrophic endoparasites that maintain a complex interaction with their host plants. Nematode effector proteins are synthesized in the oesophageal glands and are secreted into plant tissues through the stylet. To understand the function of nematode effectors in parasitic plants, we cloned predicted effectors genes from *Heterodera avenae* and transiently expressed them in *Nicotiana benthamiana*. Infiltration assays showed that HaEXPB2, a predicted expansin-like protein, caused cell death in *N. benthamiana. In situ* hybridization showed that *HaEXPB2* transcripts were localised within the subventral gland cells of the pre-parasitic second-stage nematode. *HaEXPB2* had the highest expression levels in parasitic second-stage juveniles. Subcellular localization assays revealed that HaEXPB2 could be localized in the plant cell wall after *H. avenae* infection.This The cell wall localization was likely affected by its N-terminal and C-terminal regions. In addition, we found that HaEXPB2 bound to cellulose and its carbohydrate-binding domain was required for this binding. The infectivity of *H. avenae* was significantly reduced when *HaEXPB2* was knocked down by RNA interference *in vitro*. This study indicates that HaEXPB2 may play an important role in the parasitism of *H. avenae* through targeting the host cell wall.

The cereal cyst nematode *Heterodera avenae* causes severe losses to cereal crops across the world. Grain yield losses can be as high as 30–100% in some infested fields^1^,^2^. *H. avenae* is the most economically important nematode infecting wheat (*Triticum aestivum*), oats (*Avena sativa*), barley (*Hordeum vulgare*), and rye (*Secale cereale*)^3^. *H. avenae* occurs in most wheat-growing regions of the world, including Asia, America, Australia, European and North Africa^4^. *H. avenae* has caused wheat yield losses in China^5^.

Effectors are defined as proteins and small molecules that can alter the host cell structure and function to facilitate infection or trigger defence responses^6^. Pathogens, including fungi, oomycetes, bacteria and nematodes, deliver effectors that suppress pathogen–associated molecular pattern (PAMP)-triggered immunity (PTI), the first layer of the plant immune system. Pathogen effectors can also suppress the effector-triggered immunity (ETI), the second layer of immunity which is caused by recognition of an avirulence effector by its cognate resistance protein^7^. Recent studies on effector biology from bacteria, fungi and oomycetes have provided new insights into the interactions between pathogens and hosts^8^,^9^. Similar advances have been made in the field of plant nematology^10^,^11^,^12^.

Nematode effector proteins are known to be synthesized in the esophageal glands, although other potential sources of origin also have been reported^13^,^14^,^15^. A large number of effector genes from *H. glycines* and *Globoderarostochiensis* were identified by microaspiration of the esophageal gland cell cytoplasm and sequencing of gland cell cDNA libraries^16^,^17^. The rapid advances in sequencing technology have provided tools for studying genetic resources from which candidate effector genes have been identified from a wide range of plant-parasitic nematodes. These resources include transcriptome sequences from cyst nematodes such as *H. schachtii,* and *H. glycines*^18^,^19^. The genomes of the potato cyst nematode *Globodera. pallida*^20^ and the root-knot nematodes *Meloidogyne incognita* and *M. hapla* have been sequenced^21^,^22^. Moreover, the transcriptomes of second-stage juveniles (J2s) and the female stages of *H. avenae* have also been published^23^. The transcriptomes of the *H. avenae* early parasitic stage (30 hours, 3 days and 9 days post-infection) were investigated using Illumina sequencing^24^.

Previous research showed that nematode effectors participate in the activation and suppression of host defences, plant cell wall degradation and modification, manipulation of cell fate, peptide mimicry and the regulation of plant signalling pathways^11^. The plant cell wall, a complex and dynamic association of different high-molecular-weight polysaccharides and structural, enzymatic and catalytic proteins, is the first physical barrier encountered by the nematode when parasitizing a plant^25^. Nematodes produce a range of cell wall modifying proteins that help overcome this barrier during parasitism including pectate lyase, expansin, β-1,4-endoglucanase and polygalacturonase. The β-1,4-endoglucanases, the first cell wall-degrading enzymes identified from plant-parasitic cyst nematodes, belong to glycosylhydrolase family 5 (GHF5)^26^,^27^,^28^,^29^. Pectate lyases are found in a range of cyst nematode species, such as *H. glycines*^30^, *H. schachtii*^31^, and *G. rostochiensis*^32^,^33^, while polygalacturonases have been identified in secretions of *M. incognita*^34^. A cellulose-binding protein (CBP) isolated from *H. schachtii* interacts directly with *Arabidopsis* pectin methylesterase protein 3 (PME3), activating and potentially targeting this enzyme to aid *H. schachtii* parasitism^35^.

The first nematode expansin protein (Gr-EXP1) was identified from *G rostochiensis*, and contains two distinct domains: the first domain at the N-terminus which has significant similarity with a bacterial type II carbohydrate-binding module family (CBM II), while the other domain in the C-terminus is the actual expansin and is very similar to a protein from the soil bacteria *Amycolatopsis mediterranei,* which produce aerial mycelia^36^,^37^. The cell wall extension activity of Gr-EXP1 may increase, the accessibility of cell wall components to glycanases when degrading enzymes and expansin are simultaneously secreted into host cells^36^. There is expressed sequence tag (EST) data to support the existence of expansins in other plant-parasitic nematodes^23^,^38^,^39^. The expansin-like genes *Bx-expb1* and *Bm-expb1* isolated from, *Bursaphelenchus xylophilus* and *B. mucronatus,* are similar to Gr-EXP1^40^. The expansins family in the nematodes Tylenchida and Aphelenchida is most likely of prokaryotic origin and was acquired by horizontal gene transfer^37^,^40^.

The identification and functional characterization of plant-parasitic nematode effectors should provide insight into the interaction between nematodes and plants. However, little is known about the secreted proteins produced by *H. avenae* and their functions in the parasitic process in plants. Here, we describe the identification of candidate effectors from *H. avenae*. Among the candidate proteins, we identified a new expansin protein, named HaEXPB2, that binds to cellulose via the carbohydrate-binding domain and causes cell death when transiently expressed in *N. benthamiana.*

## Results

### HaEXPB2 induces cell death in *N. benthamiana*

**Figure 1:**
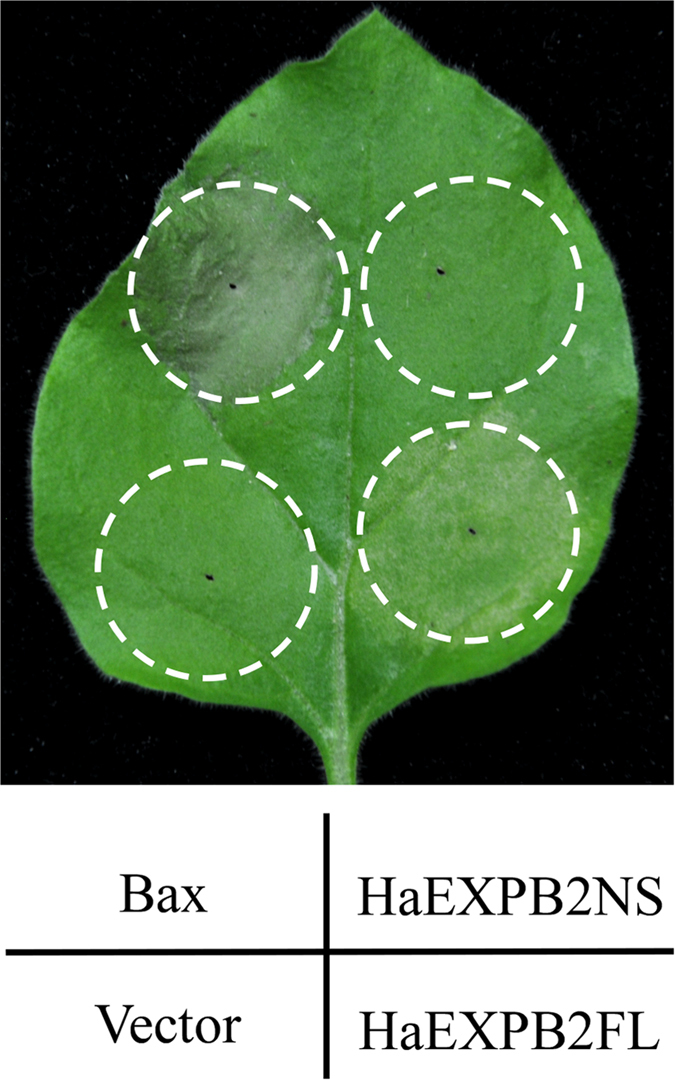
Transient expression of the full-length HaEXPB2 induces cell death in *N. benthamiana,* while the same protein lacking a signal peptide (HaEXPB2 NS) does not induce this response. Agroinfiltration was performed on the same leaf with *A. tumefaciens* carrying an empty vector control (pYBA1143), a positive control (Bax), constructs with full-length HaEXPB2 (HaEXPB2FL), and constructs lacking a signal peptide sequence HaEXPB2(HaEXPB2NS), respectively. Cell death symptoms were assessed at 5 dpi.

Analysis of available transcriptome resources for *H. avenae* revealed a total of 39 sequences; 27 of them were similar to previously identified effectors from other plant-parasitic nematodes, and 12 of them were identified as novel effectors containing predicted N-terminal signal peptides (SP) and lacking a transmembrane helix (Supplementary Table 1). All the candidate effectors were screened by transient expression assay in *N. benthamiana* leaves in order to identify candidates that induced cell death. One of the effectors caused significant cell death in *N. benthamiana*. This candidate effector gene encodes a 289 amino acid protein with 89% identity to Ha-EXPB1^41^ and was subsequently named HaEXPB2 (GenBank KX369393). The full-length HaEXPB2 sequence induced noticeable cell death, while HaEXPB2 lacking the signal peptide sequence did not cause any cell death in *N. benthamiana* (Fig. 1), suggesting that the protein needs to be exported in the apoplast in order to generate the cell death phenotype. The cell death caused by HaEXPB2, which appeared 3–4 days after agro-infiltration, was not as strong as that induced by Bax. The empty vector control did not induce cell death at any time. Because HaEXPB2 is very similar to Ha-EXPB1, our results also confirmed that Ha-EXPB1 induces cell death in *N. benthamiana* (Fig. S1). The results suggest that Ha-EXPB1 and HaEXPB2 cause significant cell death when transiently expressed in *N. benthamiana*.

### Sequence and structural analysis of HaEXPB2

**Figure 2:**
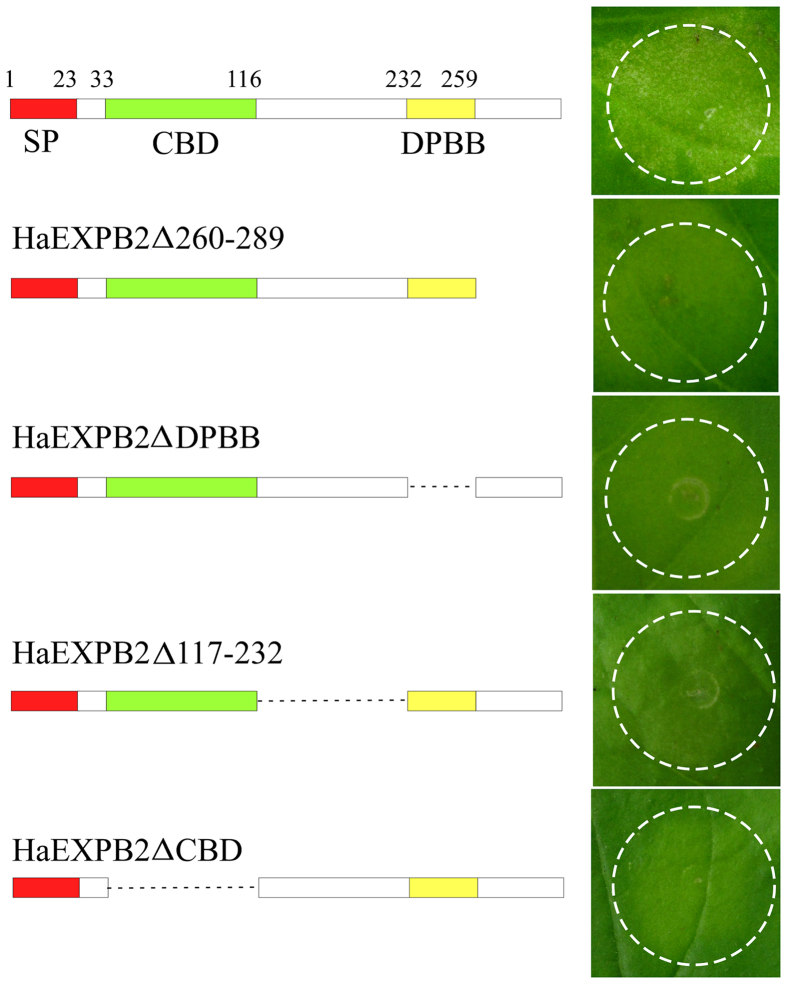
Structural analysis of the regions of HaEXPB2 responsible for inducing cell death. Schematic views of HaEXPB2 and their deletion mutants are shown on the left. The predicted domains or motifs of HaEXPB2 are represented as colored rectangles. The deleted regions are represented as dotted lines. HaEXPB2 and their deletion mutants were transiently expressed in *N. benthamiana* leaves. Each experiment was repeated at least three times with similar results.

**Figure 3:**
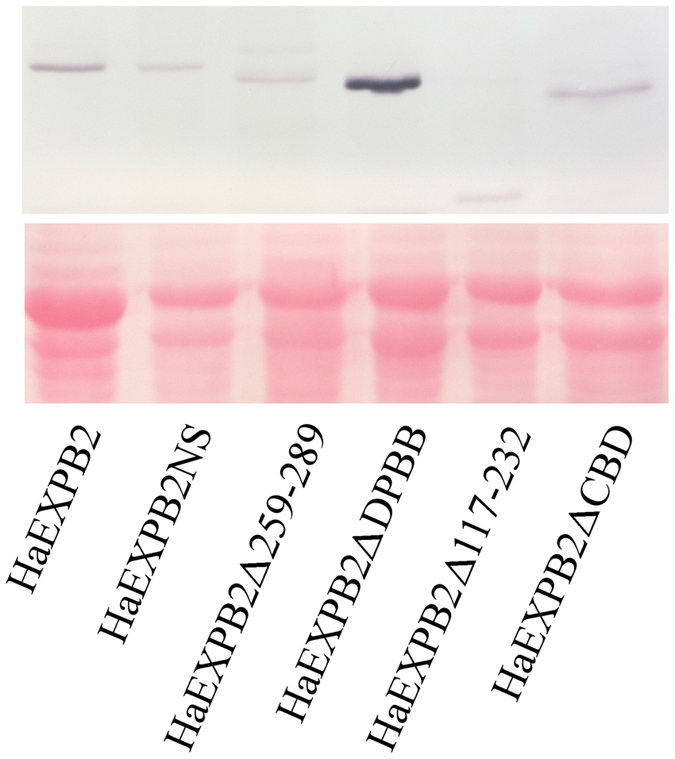
Western blot analysis of HaEXPB2 and HaEXPB2 deletion series. The anti-HAantibody was used to detect the accumulation of fused proteins in the leaves of *N. benthamiana* plants. Ponceau S staining (lower panel) was used to show equal loading.

Sequence analysis showed that HaEXPB2 was similar to expansins and expansin-like proteins from plant-parasitic nematodes and contained a signal peptide (residues 1–23), a carbohydrate-binding domain (CBD) (residues 33–116) and an β-expansin domain (DPBB) (residues 232–259) (Fig. 2). To identify the domains responsible for inducing cell death, a series of deletion variants were generated to perform functional analysis. HaEXPB2Δ260–289 mutants lacking the C-terminus (residues 260–289) failed to induce cell death. The variant protein HaEXPB2ΔDPBB lacking the β-expansin domain (residues 232–259), also did not cause cell death in *N. benthamiana*. In addition, HaEXPB2ΔCBD which lacks the CBD, and a deletion mutant (HaEXPB2Δ117–232) lacking the fragment from residues 117–232 failed to induce cell death in *N. benthamiana*. All effector fusion proteins were expressed in the leaves of *N. benthamiana*, as evidenced by western blotting (Fig. 3). This indicates that the entire HaEXPB2 protein is necessary for inducing cell death in *N. benthamiana*.

### Localization of *HaEXPB2* transcripts and developmental expression pattern of *HaEXPB2*

**Figure 4:**
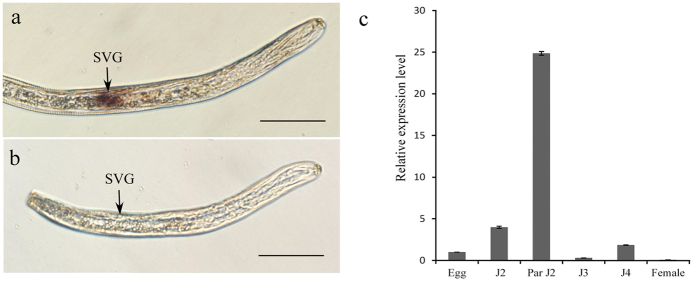
*In situ* hybridization and developmental expression pattern analysis of *HaEXPB2*. (**a**) Signal of antisense *HaEXPB2* DIG-labeled cDNA probes localized within the subventral glands (SVGs). (**b**) Sense probes as a negative control. Scale bar = 20 μm. (**c**) Developmental expression pattern of *HaEXPB2*. The relative expression level of *HaEXPB2* was quantified using qPCR for six different *H. avenae* stages. The fold change values were calculated using the 2-ΔΔCt method and presented as the change in mRNA level in various nematode developmental stages relative to that of egg. Each column represents the mean of three independent assays with standard deviation. J2: pre-parasitic second-stage juvenile; parJ2, J3 and J4: parasitic second-, third- and fourth-stage juvenile, respectively.

We performed *in situ* hybridization to analyse the spatial expression profile of *HaEXPB2* in J2s. The digoxigenin-labeled *HaEXPB2* antisense probes were specifically detected in the subventral gland cells of *H. avenae* (Fig. 4a), while no signal was observed using sense probes (Fig. 4b). The expression pattern of *HaEXPB2* in different developmental stages may indicate the stage of the parasitic process that the protein is important for. We therefore performed real-time PCR to determine the expression pattern of *HaEXPB2*. In the six developmental stages tested, the expression level of *HaEXPB2* in the parasitic J2 stage was higher than in the other stages tested (Fig. 4c). These results indicate that HaEXPB2 is synthesized in the esophageal glands of *H. avenae* and plays a role in the early parasitic process.

### HaEXPB2 is localized in the plant cell wall

**Figure 5:**
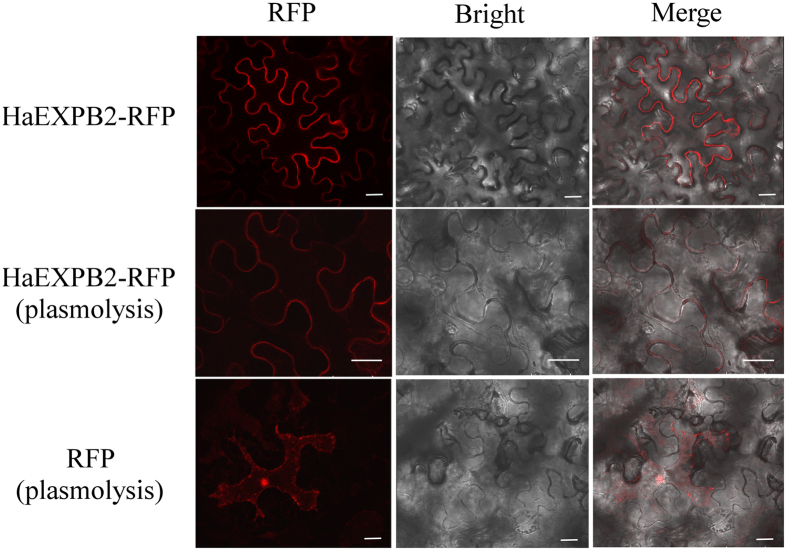
Subcellular localization of transiently expressed HaEXPB2-RFP fusions in *N. benthamiana* leaves. The upper panel was HaEXPB2-RFP in normal cell, the middle panel was HaEXPB2-RFP in plasmolysed cell, and the lower panel was control empty vector in plasmolysed cell. Scale bar = 20 μm.

To investigate the subcellular localization of HaEXPB2 in plant cells, we fused the reporter protein RFP to the C-terminus of the HaEXPB2 protein. The RFP signal from the 35S::HaEXPB2-RFP fusion expressed in *N. benthamiana* was observed at the cell edge. To distinguish labelling in the cell wall and the plasma membrane, the plant cells were plasmolysed by treating with glycerol. The 35S:: HaEXPB2-RFP signal was detected in the cell wall with plasmolysis, but not in the plasma membrane (Fig. 5). The subcellular localization of HaEXPB1 was the same as that of HaEXPB2 in the plant cell wall (Fig. S2).

**Figure 6:**
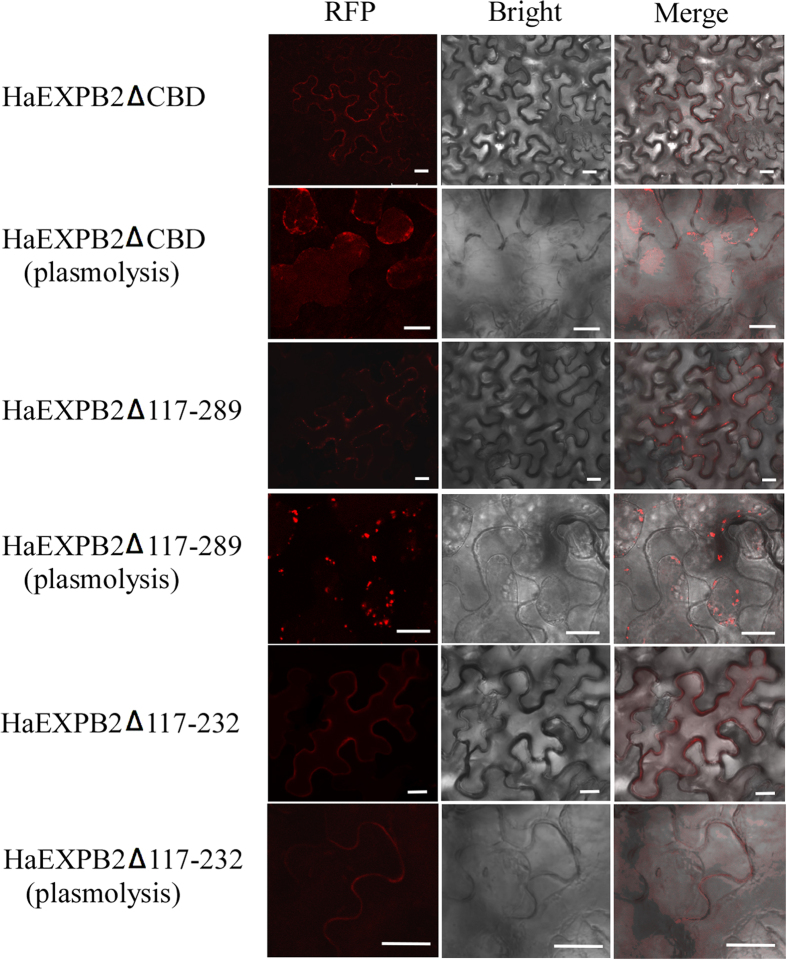
Subcellular localization of transiently expressed HaEXPB2 deletion mutants in *N. benthamiana* leaves. HaEXPB2ΔCBD, HaEXPB2Δ117-232 and HaEXPB2Δ117-289 were fused to N-terminal of RFP and expressed in *N. benthamiana.* All of them were observed in normal and plasmolysed cells. Scale bar = 20 μm.

We then fused a series of HaEXPB2 deletion variants to RFP to test the localisation of the CBD domain. When the CBD was removed, the resulting HaEXPB2ΔCBD variant was localized in the plasma membrane but not in the cell wall (Fig. 6). Consequently, to confirm the function of CBD, a mutated HaEXPB2Δ117–289 fusion protein that only contained the signal peptide and the CBD was created for a subcellular localization assay. Unexpectedly, the fragment with signal peptide and CBD was not localized in the cell wall. The deletion mutant HaEXPB2Δ117–232, lacking a central fragment, was still localized in the cell wall (Fig. 6). These data suggest that regions in the N-terminus and C-terminus are necessary for the localization of HaEXPB2 in the plant cell wall.

### HaEXPB2 binds to cellulose via its carbohydrate-binding domain

**Figure 7:**
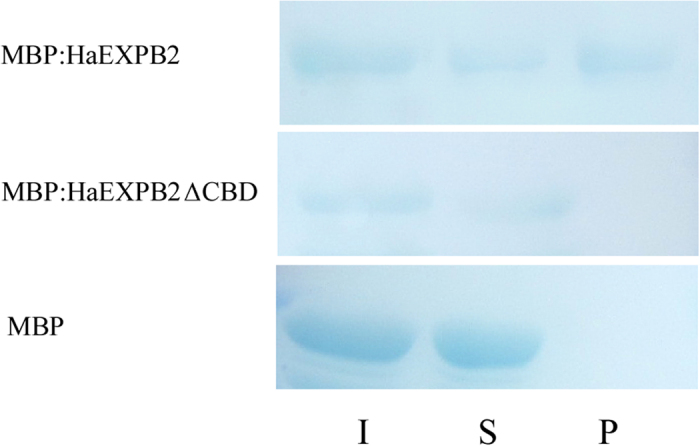
Cellulose binding activity of HaEXPB2. The purified protein MBP, MBP: HaEXPB2, MBP: HaEXPB2ΔCBD were mixed with cotton linters separately. The mixture of protein and substrate was centrifuged to obtain supernatant (S) and pellet (P) fraction. Input (I) indicates the mixture of protein and substrate before separation. The presence of the HaEXPB2 with CBD is required for the protein to bind to cellulose.

To investigate whether HaEXPB2 has cellulose binding activity, we produced fusions of maltose-binding protein to HaEXPB2 (MBP:HaEXPB2) and HaEXPB2 lacking the carbohydrate-binding domain (MBP:HaEXPB2ΔCBD). These proteins were purified and incubated with microcrystalline cellulose to detect whether they could bind to it. MBP:HaEXPB2 was detected in the microcrystalline cellulose substrate pellet, but MBP:HaEXPB2ΔCBD and the negative control MBP were not found in the pellet (Fig. 7). These results show that HaEXPB2 has a function in the cell wall and that its binding to cellulose is mediated through the carbohydrate-binding domain.

### *HaEXPB2* RNAi *in vitro* reduces *H. avenea* infectivity

**Figure 8:**
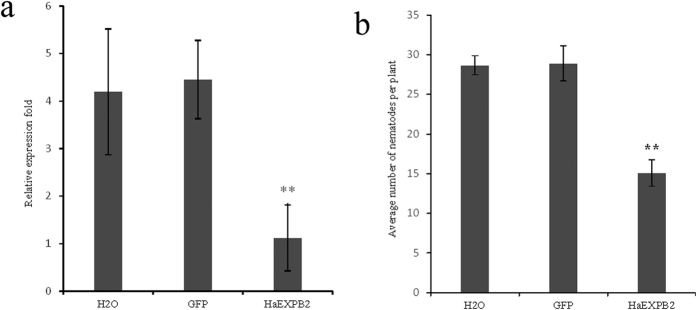
Effect of RNAi of *HaEXPB2 in vitro* on *H. avenae* susceptibility. (**a**) Relative expression level of *HaEXPB2* in nematodes treated with *HaEXPB2*dsRNAs, *gfp* dsRNA and water. (**b**) The number of nematodes in wheat roots at 10 dpi. Each column represents the mean of three independent assays with standard deviation. “**” indicates significant differences based on Student’s t-test (P < 0.01).

In this study, RNAi was used to investigate the function of HaEXPB2 in parasitism. The *HaEXPB2* transcripts expressed in juveniles were detected by real-time PCR after soaking in dsRNA for 24 hours. The mRNA expression level of *HaEXPB2* in nematodes treated with *HaEXPB2* dsRNA dramatically decreased by 73.5% compared with that in nematodes treated with water (Fig. 8a). Therefore, *HaEXPB2* was effectively silenced by soaking in dsRNA. The infectivity of *H. avenea* was tested 10 days after inoculating J2s by soaking with the *HaEXPB2* dsRNAs, *gfp* dsRNA or water. The number of nematodes inside the roots was decreased by 53.4% compared with the control water treatment (Fig. 8b). The number of nematodes after treatment with *gfp* dsRNA was not significantly different compared with the control. Our results indicate that HaEXPB2 plays an important role in facilitating parasitism in the early stage of *H. avenea.*

## Discussion

Identification and functional analysis of plant-parasitic nematode secreted proteins involved in key processes of the nematode-host interaction will provide insights into the mechanisms of successful parasitism. From 39 *H. avenae* putative secreted proteins, we successfully identified HaEXPB2, an expansin protein, which caused cell death in the plant *N. benthamiana.* The secreted proteins from plant pathogens have been tested directly for the induction or suppression of plant defence responses through its expression in plant leaves, as an alternative to obtain stable transgenic plants. In this way, virulence or avirulence effectors that can induce cell death in plants have been identified from several eukaryotic plant pathogens^42^,^43^,^44^,^45^. An expansin protein from *G. rostochiensis*, GrEXPB2, which lacks a carbohydrate-binding domain (CBD) found in expansins and expansin-like proteins, also induced chlorosis in *N. benthamiana* and cell death in tomato and potato plant^46^. The cell death symptoms induced by GrEXPB2 were eliminated when the signal peptide was deleted. Consistent with this, HaEXPB2 lacking the signal peptide also failed to induce cell death, indicating that this response is only induced when the protein is located outside the cell. GrEXPB1 and GrEXPB2 belong to the same expansin-like family of proteins, but GrEXPB1 does not induce any cell death phenotype. Our studies showed that Ha-EXPB1, which has 89% identity to HaEXPB2, also caused a significant cell death in *N. benthamiana*. The subcellular localization of Ha-EXPB1 was the same as that of HaEXPB2, with both found in the cell wall. Therefore, our result showed that Ha-EXPB1and HaEXPB2 may have the same function when the nematode parasitizes plants. To determine whether HaEXPB2 affects root development, HaEXPB2-over-expressing *Arabidopsis thaliana* lines were generated through transformation. The root lengths of homozygous lines were not significantly increased compared with the wild-type (Fig. S3). The same morphological phenotype demonstrated that HaEXPB2 has no function in root development.

Nematode effectors are synthesized in the esophageal glands of the nematode and delivered into the host plant tissue or cells through the stylet^15^. The first identified nematode expansin protein (Gr-EXP1) is produced in the subventral oesophageal glands of pre-parasitic second stage juveniles^36^. *Ha-expb1*,-an expansin gene cloned from *H. avenae*, is also expressed in the subventral glands. The transcripts of *Ha-expb1* were higher in the early juvenile stages^41^. In our study, *in situ* hybridization showed that HaEXPB2 was synthesized in the subventral esophageal glands, and that the expression of *HaEXPB2* was the highest in the parasitic J2 stage. The results provide evidence that expansin proteins are secreted proteins that play important roles in the early stages of parasitism.

To study the importance of HaEXPB2 in the parasitism of *H. avenae,* genes were knocked down by RNA interference. Previous research demonstrated that RNA interference by soaking in dsRNA is effective to analyse the function of pathogenicity genes in cyst nematodes^47^. In our results, the penetration rate was reduced by more than 50% when *HaEXPB2* was knocked down by soaking in dsRNA. However, the numbers of cyst produced by the nematodes treated with *HaEXPB2*dsRNAs, *gfp* dsRNA and water showed no significant difference. This result is similar to the cell-wall-modifying gene *Ha-eng-2*; silencing *Ha-eng2* by RNAi caused a significant reduction in infection^48^. Knocking down the β-1,4-endoglucanases genes *Gr-eng1. Gr-eng2*, *Gr-eng3*, *Gr-eng4* also reduced the infectivity of *G. rostochiensis*^49^,^50^. Additionally, *Ha-eng2*, *Gr-eng3* and *Gr-eng4* encode active cellulases *in vitro*^48^,^50^. Therefore, the functions of HaEXPB2 may be to facilitate infection by modifying the cell wall in the early stage.

Understanding the destination of effector proteins can help improve the understanding of their function in the interaction with the host. However, where exactly the effector proteins are deposited in the host cell, (directly to the cytoplasm or the apoplastic space) is not very clearly^11^. Ultrastructural analysis of root cortical cells shows a small pore at the stylet orifice, supporting the idea effector proteins are delivered directly into the host cell cytoplasm^51^.A *M. incognita* effector protein, 7H08, containing nuclear localization signals (NLSs), was localized to the nuclei of plant cells, and showed transcriptional activation activity in both yeast and plant systems^52^. The 10A07 effector from *H. schachtii* is phosphorylated by a plant kinase in the cytoplasm and then translocated to the nucleus, interacting with the transcription factor IAA16 to promote parasitism^53^. Cell wall localization of HaEXPB2 provides direct evidence that nematode expansin proteins have an important function in the plant cell wall. Previous immunolocalization shows that the plant apoplast is an important destination compartment where effector proteins are delivered, including cell-wall-modifying proteins (Mi-PEL3, CBM2-bearing proteins)^54^. HaEXPB2, as a cell-wall-modifying protein, contains a CBM2 and should be deposited in the apoplast when it is secreted by nematodes. The calreticulin (CRT) Mi-CRT from M. incognita is secreted into plant tissues via the stylet, and accumulates at the cell wall of giant cells^55^. Then, Mi-CRT without the signal peptide is located in the cytoplasm, while the localization of full-length Mi-CRT is located in the endoplasmic reticulum (ER) and Golgi, indicating that the signal peptide is functional in plant cells, and Mi-CRT with the signal peptide entered the plant secretory pathway^56^. Our results validated the hypothesis that HaEXPB2 containing the signal peptide can function in the apoplast, and the signal peptide could target the protein to the apoplast when HaEXPB2 was overexpressed in *N. benthamiana*.

Cell-wall-modifying proteins that have been found in the secretions of plant-parasitic nematodes contain a carbohydrate-binding domain (CBD), which is predicted to bind specifically to cellulose^57^,^58^. The CBD of expansin shows significant similarity to the carbohydrate-binding module (CBM) family II of endoglucanases, and its DPBB was similar to the expansin-like protein (PPAL) from plants^36^. The CBDs were defined as non-catalytic polysaccharide-recognizing modules of glycoside hydrolases because they were the first examples of these protein domains that bound crystalline cellulose as their primary ligand^59^. CBMs have three general functions: a proximity effect, a targeting function and a disruptive function^60^. Gr-EXP1 contains a carbohydrate-binding domain and a β-expansin domain and showed significant cell wall extension activity on wheatbut exhibited no other enzymatic activity^36^. A swollenin protein from *Trichoderma asperellum*, comprising an N-terminal CBD and a C-terminal expansin-like domain, increased plant root colonization. The CBD was indispensable for the full protein activity of the swollenin protein^61^. However, our results showed that HaEXPB2Δ117-289, only containing CBD, failed to be targeted the cell wall, while HaEXPB2Δ117–232, with both the CBD and DPBB domains, was localized in the cell wall. We presume that the DPBB plays a role when HaEXPB2 binds to polysaccharides. Previous study has shown that linker stiffness and length are important modulators of cellulase activity^62^,^63^. HaEXPB2Δ117-232 without linker region failed to induce cell death. It seems that that the linker region (117–232) affects the structure and function of expansin protein. In addition to exhibiting cell-wall localization, HaEXPB2 was shown to possess carbohydrate-binding activity *in vivo,* and it was found that the CBD was required for this activity. Thus, it is likely that the CBD allows the expansin to bind to polysaccharides, bringing the functional domain close to the substrate. Further studies would be required to determine how the expansin proteins target the cell wall to facilitate nematodes parasitism.

Specific pathogen effectors can be recognized by specific nucleotide-binding leucine-rich repeat (NLR) receptors and elicit cell death characteristic of a hypersensitive response (HR). For example, the effector protein RBP-1 from *G. pallida* was reported to elicit cell death through its recognition by the NB-LRR protein Gpa2^64^. In this study, we could not conclude whether HaEXPB2 induced cell death due to recognition by NLR proteins in the plant innate immune system. Interestingly, the CBDs are identified a novel class of molecular patterns that may function via their interaction with the cell wall^61^,^65^. The nematode expansin protein Gr-EXP1 showed extension activity on wheat and cucumbers, while GrEXPB2 induced chlorosis in *N. benthamiana* and a necrotic response in tomato and potato plants^36^,^46^. We assume that the cell death caused by HaEXPB2 may have occurred as a result of changes to the cell wall.

Our result indicated that HaEXPB2 is involved in the process of penetration and migration of *H. avenae* by binding to the cell wall directly, but the mechanism is not clear. This process may be perceived by the plant and may contribute to the activation of host defence responses. The cell wall is an important battlefield in the molecular dialogue between the nematode and the host.

## Materials and Methods

### Nematode material

*Heterodera avenae* was cultured on wheat (*Triticum aestivum* cv. Wenmai 19) in a growth chamber^66^. The temperature of the seedling cultures was maintained at 16 °C for the first week and then at 22 °C for the remaining growth period. The mature cysts were extracted from the soil and hatched to pre-parasitic J2s after incubating at 4 °C for 6 weeks. Wheat roots infected with *H. avenae* J2s were harvested at 5, 10, 20 and 30 days post-inoculation (dpi), and different parasitic stages of *H. avenae* were collected by digesting the roots at 28 °C with shaking at 160 rpm in a 6% cellulose and pectinase solution overnight^67^. The pJ2 were obtained from 5 dpi directly. The J3 and J4 were obtained under the microscope from 20 dpi and 30 dpi separately. Some of the pJ3 were picked up from 10 dpi. Adult females were hand-picked from root surfaces under a dissecting microscope at 40 dpi.

### Sequence analyses and construction of vectors

The putative effectors were selected from *H. avenae* transcript libraries. Signal peptides were predicted using signalP 4.1 (http://www.cbs.dtu.dk/services/SignalP/)^68^, and putative transmembrane helices were predicted by TMHMM Server v.2.0

(http://www.cbs.dtu.dk/services/TMHMM/). The open reading frames were predicted by ORF Finder (http://www.ncbi.nlm.nih.gov/projects/gorf/gorf.html). The coding sequences of candidate genes were amplified with high-fidelity DNA polymerase (TaKaRa, Japan). Then, the coding sequences were cloned into the plant expression vector pYBA1143 using restriction enzyme sites. The HaEXPB2 coding sequences with and without signal peptide were amplified using gene-specific primers containing BamHI and HindIII restriction enzyme sites. The HaEXPB2Δ260-289 sequence was amplified using HaEXPB2F (*Bam*HI)/HaEXPB2R1 (*Hind* III). HaEXPB2Δ117-289 sequence was amplified using HaEXPB2F (*Bam*HI)/HaEXPB2R3 (*Hind* III). The sequence of HaEXPB2ΔCBD, HaEXPB2ΔDPBB, and HaEXPB2Δ117-232 were amplified using HaEXPB2-4R/HaEXPB2-4F, HaEXPB2-5R/HaEXPB2-5F, HaEXPB2-6R/HaEXPB2-6F and HaEXPB2F (*Bam*HI)/HaEXPB2R1 (*Hind* III) by overlapping PCR, respectively (Supplementary Table 2). The resulting amplified fragments were cloned into the respective sites in the modified pYBA1143 vector, which contained an HA tag. All constructs in this study were confirmed by sequencing.

### *Agrobacterium*-mediated transient expression

The pYBA1143-based constructs of *H. avenae* effector genes were transformed into the *Agrobacterium tumefaciens* strain EHA105. The bacterial cells were collected at 4,000×g for 10 minutes, re-suspended in MES buffer (200μM acetosyringone, 10 mM MgCl_2_ and 10 mM MES, pH 5.6) after shaking for 18 hours at 28 °C at 200 rpm. The bacterial suspensions were adjusted to an OD600 of 1.0 and mixed with P19 (an RNA-silencing suppressor) at 1:1. Then, the mixture was kept at room temperature and in the dark for 3 hours. The *A. tumefaciens* strain carrying the constructs were infiltrated into the leaves of *N. benthamiana* grown in a growth room for 6–8 weeks at approximately 22 °C with a 14 hours light/10 hours dark cycle. The empty pYBA1143 vector and Bax were used as a negative and positive control, respectively^44^.

The overexpression construct pYBA1143::HaEXPB2 was transformed into *Agrobacterium tumefaciens* strain GV3101. Transgenic *Arabidopsis thaliana* carrying the pYBA1143::HaEXPB2 construct was generated by the floral dip method. The seeds were germinated on Murashige and Skoog (MS) medium. The homozygous transgenic plants from T3 10 days after germination were used in this study.

### *In situ* hybridization and real time quantitave PCR

*In situ* hybridization was performed to confirm the expression site of *HaEXPB2* as previously described with some modifications^69^. The specific primers of *HaEXPB2* were used to synthesise digoxigenin (DIG)-labelled sense and antisense cDNA probes (Roche Diagnostics, Mannheim, Germany) by asymmetric PCR. Hybridization signals within the nematodes were detected by alkaline phosphatase immunostaining and specimens were observed with an Olympus IX71 microscope.

Total RNA was isolated from pre-parasitic J2, parasitic J2, J3, J4, adult females and eggs using TRIzol (Invitrogen, Carlsbad, CA, USA) according to the manufacturer’s instructions. All total RNA samples were treated with RNase-free DNase1 to remove DNA contamination. First-strand cDNA synthesis was conducted using the SuperScript™ III First-Strand Synthesis System for RT-PCR kit (Invitrogen). The primers GAPDH-qS1/GAPDH-qAS1 targeted the endogenous control gene GAPDH-1^70^. The PCR reactions were run using SYBR Premix ExTaq (TaKaRa, Japan) in an ABI Prism 7500 instrument (Applied Biosystems, USA). Quantification of the relative changes in gene expression was performed using the 2^−ΔΔCT^ method with actin as the endogenous control gene. At least three independent experiments, each with three technical replicates of each reaction, were performed.

### Subcellular localization

The coding sequences of HaEXPB1, HaEXPB2, HaEXPB2ΔCBD, HaEXPB2Δ117–289 and HaEXPB2Δ117–23 were cloned into the pYBA1137 vector which contained the RFP gene. *A. tumefaciens* EHA105 carrying the fused constructs were allowed to infiltrate the leaves *N. benthamiana* as previously described (part of *Agrobacterium*-mediated transient expression). The subcellular localization of the fused proteins were observed using laser confocal fluorescence microscopy 3 days after infiltration^71^. To distinguish the cell wall and the plasma membrane, the leaf cells were plasmolysed by infiltrating 30% glycerol solution for 10 min before observation^72^. All samples were observed with a Leica TCSSL microscopy.

### Binding activity assay

The binding of recombinant proteins to microcrystalline cellulose substrate was performed as described previously^73^. The HaEXPB2 and HaEXPB2ΔCBD sequences were cloned into the pMAL-c2 vector and transformed into the *Escherichia coli* strain BL21 (DE3). IPTG was added to a final concentration of 0.5 mM to induce protein expression. The proteins MBP: HaEXPB2 and MBP: HaEXPB2ΔCBD were purified using amylose resin (NEB). The purified proteins were incubated with 1% cotton linters (Sigma) in 50 mM sodium acetate buffer (pH 5.0) at room temperature for 1 hour and centrifuged at 15,000 ×g for 2 minutes. The supernatants were removed and the pellet was washed with the same buffer two times. All samples were separated on 15% SDS-PAGE and stained with Coomassie Brilliant Blue.

### Western blot analysis

The *N. benthamiana* leaf samples were harvested 4 days after infiltration. Total protein was extracted from plant tissue powder using phenol solution (0.5 M Tris pH 9.4, 50 mM EDTA, 0.7 M sucrose, 0.1 M KCl containing 2% Mercaptoethanol and protease inhibitors cOmplete (Roche)). The protein concentration was measured using a Bradford assay following the manufacturer’s instructions (Bio-rad). Equal amounts of protein samples were separated on Tris-Glycine SDS-PAGE gels and transferred to nitrocellulose membranes using an iBlot (semi-) dry blotting system from Life Technologies. The blotting membrane was blocked with 5% BSA (bovine serum albumin) in TBST solution (50 mM Tris-HCl, pH 7.5, 150 mM NaCl, 0.05% Tween20) for 1 hour at room temperature. The membrane was incubated with a 1:5000 dilution anti-HA primary antibody (Sigma) for 3 hours at room temperature. The membrane was washed with TBST solution and incubated with a 1:30000 dilution of anti-mouse IgG (Fc specific)-alkaline phosphatase antibody (Sigma). The signal was detected using an AP conjugate substrate kit (Bio-rad). Western blots were replicated using protein extracts from three independent isolations.

### RNA interference *in vitro* and inoculation

The fragment (76–319 bp) of *HaEXPB2* was designed as the target. The *HaEXPB2* dsRNA and *gfp* dsRNA were synthesized and purified with a MEGAscript RNAi Kit (Applied Bio-systems, Austin, TX, USA) with the primers DsHaEXPB2F/DsHaEXPB2FR, and GFPT7F/GFPT7R, respectively.The RNAi soaking was performed following Urwin *et al.*^74^. Approximately 10,000 freshly hatched J2s were soaked in M9 buffer that included 50 mM octopamine, 3 mM spermidine, 0.05% gelatin, and 2 mg/ml dsRNA at room temperature in the dark on a rotator. The treated J2s were washed at least three times with nuclease-free water to remove the external dsRNA. Total RNA was extracted from the treated J2s for real-time PCR analysis.

One-week-old *T. aestivum* cv. Wenmai 19 were inoculated with soaked J2s (100 J2s per plant). The roots were harvested at 10 dpi for acid fuchsin staining and were then observed and counted under a microscope^75^. The number of cysts on the infected plant root was analysed at 40 dpi inoculation. The experiment was repeated in triplicate and results were analysed using Duncan’s multiple range test.

## Additional Information

**How to cite this article**: Liu, J. *et al.* Molecular Characterization of A Novel Effector Expansin-like Protein from *Heterodera avenae* that Induces Cell Death in *Nicotiana benthamiana.* *Sci. Rep.* **6**, 35677; doi: 10.1038/srep35677 (2016).

**Publisher’s note**: Springer Nature remains neutral with regard to jurisdictional claims in published maps and institutional affiliations.

## Supplementary Material

Supplementary Information
