# Supplementary material for: Molecular Characterization of A Novel Effector Expansin-like Protein from Heterodera avenae that Induces Cell Death in Nicotiana benthamiana
Source: Sci Rep. 2016 Nov 3;6:35677. doi: 10.1038/srep35677 (PMC5093861; doi:10.1038/srep35677)
Supplement: Supplementary Information [file srep35677-s1.pdf]

Molecular Characterization of A Novel Effector Expansin-like Protein from *Heterodera avenae* that Induces Cell Death in *Nicotiana benthamiana*

Jing Liu[1,2†], Huan Peng[1†], Jiangkuan Cui[1], Wenkun Huang[1], Lingan Kong[1], Jihong Liu Clarke[3], Heng Jian[2], Guo Liang Wang[1,4] , Deliang Peng[1*]

Supplementa Table1. List of 39 *Heterodera avenae* candidate effectors

Unigene ID	Hit gene	Hit Genbank accession	E-value	Bitscore
comp34893_c0_seq1	Globodera rostochiensis secreted SPRY domain-containing protein 18mRNA, complete cds	JX026919	1E-30	112
comp61735_c0_seq1	Globodera pallida clone GPS9-13 RBP-1 protein (rbp-1) mRNA, complete cds	FJ882995	9E-32	117
comp52750_c1_seq1	Globodera pallida mRNA for putative SEC-2 protein mRNA, complete cds	Y09293	1E-19	81.6
comp62055_c0_seq3	Ditylenchus destructor beta-1,4-endoglucanase (eng-2) mRNA, complete cds	FJ374266	2E-92	280
comp53548_c0_seq2	Dictyocaulus viviparus cholesterol acyltransferase mRNA, complete cds	JX026919	4E-34	123
comp64895_c3_seq2	Heterodera avenae acid phosphatase precursor (Ha-acp-1) mRNA, complete cds	JQ341956	5E-135	399
comp63317_c1_seq1	Heterodera avenae expansin B1 mRNA, complete cds	HQ386233	0	515
comp64381_c2_seq1	Heterodera avenae pectate lyase 1 (PEL1) mRNA, complete cds	GQ998895	0	646

comp50047_c0_seq2	Heterodera glycines hypothetical esophageal gland cell secretory protein 4 (hsp4) mRNA, complete cds	AF273731	4E-70	224
comp57204_c0_seq1	Heterodera glycines beta-1,4-endoglucanase precursor (GR-eng1)	AF004523	4E-169	486
comp34808_c0_seq1	Heterodera glycines beta-1,4-endoglucanase-4 mRNA, complete cds	AY043224	2E-85	261
comp62243_c0_seq1	Heterodera glycines beta-1,4-endoglucanase-4 mRNA, complete cds	AY043224	7E-163	458
comp60159_c1_seq1	Heterodera glycines cellulase ENG-5 gene, complete cds	AY336935	1E-162	458
comp77730_c0_seq1	Heterodera glycines chitinase mRNA, complete cds	AF468679	3E-178	499
comp67130_c0_seq1	Heterodera glycines hypothetical esophageal gland cell secretory protein 6 (hsp6) mRNA,	AF273733	7E-150	420
comp65961_c0_seq1	Heterodera glycines putative gland protein G12H04 mRNA, complete cds	AF490244	1E-38	143
comp62389_c0_seq3	Heterodera glycines putative gland protein G26D05 mRNA, complete cds	AY101191	1E-168	473
comp63301_c0_seq1	Heterodera glycines putative gland protein G27D09 mRNA, complete cds	AY101190	2E-10	55.5
comp47703_c0_seq1	Heterodera schachtii annexin 4F01 mRNA, complete cds	FJ768021	1E-73	232
comp55464_c0_seq1	Heterodera schachtii annexin 4F01 mRNA, complete cds	FJ768021	2E-165	465
comp52052_c0_seq1	Meloidogyne incognita calreticulin mRNA, complete cds	AF402771	2E-92	289

comp64559_c0_seq1	Meloidogyne incognita glutathione S-transferase-1 (gsts-1) mRNA, complete cds	EF429119	1E-48	166
comp35613_c0_seq1	Meloidogyne incognita mRNA for putative aspartyl protease precursor (asp2 gene)	FN179274	3E-27	109
comp64193_c0_seq1	Meloidogyne incognita mRNA for putative aspartyl protease precursor (asp2 gene)	FN179274	1E-26	107
comp53976_c0_seq2	Meloidogyne incognita putative esophageal gland cell secretory protein 21 (msp21) mRNA,	AY134440	2E-23	97.8
comp69427_c0_seq1	Meloidogyne incognita putative esophageal gland cell secretory protein 21 (msp21) mRNA,	AY134440	5E-09	53.1
comp67352_c0_seq1	Meloidogyne incognita putative esophageal gland cell secretory protein 26 (msp26) mRNA,	AY135362	5E-84	258
comp62468_c0_seq1	Hypothetical protein			
comp63170_c0_seq2	Hypothetical protein			
comp64896_c2_seq2	Hypothetical protein			
comp64165_c2_seq9	Hypothetical protein			
comp63625_c0_seq1	Hypothetical protein			
comp60707_c0_seq2	Hypothetical protein			
comp34853_c0_seq1	Hypothetical protein			

comp67308_c0_seq1	Hypothetical protein			
comp63997_c0_seq1	Hypothetical protein			
comp62948_c2_seq1	Hypothetical protein			
comp55920_c0_seq3	Hypothetical protein			
comp59782_c0_seq3	Hypothetical protein			

Supplemental Table2. List of primers in this study.

Primer Name	Primer sequences (5'-3')
HaEXPB2F	ATGTATACATTTCTCTCTTTGTTTG
HaEXPB2R	TCAGCATGGTTTAAGGTTTG
HaEXPB2F(*Bam*HI)	CGGGATCC ATGTATACATTTCTCTCTTTGTTTG
HaEXPB2FNS(*Bam*HI)	CGGGATCC ATGGATGTCACGGCAACG
HaEXPB2R (*Hind*III)	CCCAAGCTT GCATGGTTTAAGGTTTG
HaEXPB2R1 (*Hind*III)	CCCAAGCTT AAAAGCAGAAGTGGACAAGTCC
HaEXPB2R3 (*Hind*III)	CCCAAGCTT AATCAATTGGACGGTCGG
HaEXPB2-4R	TCAATCAAGACATCGGCCAAAACAATG
HaEXPB2-4F	GGCCGATGTC TTGATTGAGACTAAATATTTCG
HaEXPB2-5F	TGACTACATCTTTTTGGAGCCTG
HaEXPB2-5R	CAAAAAGATGTAGTCAATCTTTATGCAA A

HaEXPB2-6R	TTCCCCTTTTGGACGGTCGG
HaEXPB2-6F	GTCCAAAAGGGGAAAACTTT
HaEXPB2QF	CACGGCAACGCTCAAGACAC
HaEXPB2QR	GCCCTGAACATTGATTCCACC
GAPDH-qS1	AGCGGCACAGAACATCATCC
GAPDH-qAS1	GGTCCTCCGTGTAGCCCAAA
SH- HaEXPB2F	AAAATGGTGGAATCAATGTTCAGGG
SH- HaEXPB2R	CAGTCAAAGTTTTCCCCTTGTAGTCA
DsHaEXPB2F	TAATACGACTCACTATAGGGACGGCAA CGCTCAAGACACT
DsHaEXPB2FR	TAATACGACTCACTATAGGGCCTGAACA TTGATTCCACCATTT
GFPT7F	TAATACGACTCACTATAGGGGCACTACT GGAAAACTACCTG
GFPT7R	TAATACGACTCACTATAGGGGCACGTGT CTTGTAGTTCC
UBP22-F	GCCAAAGCTGTGGAGAAAAG

UBP22-R	TGTTTAGGCGGAACGGATAC

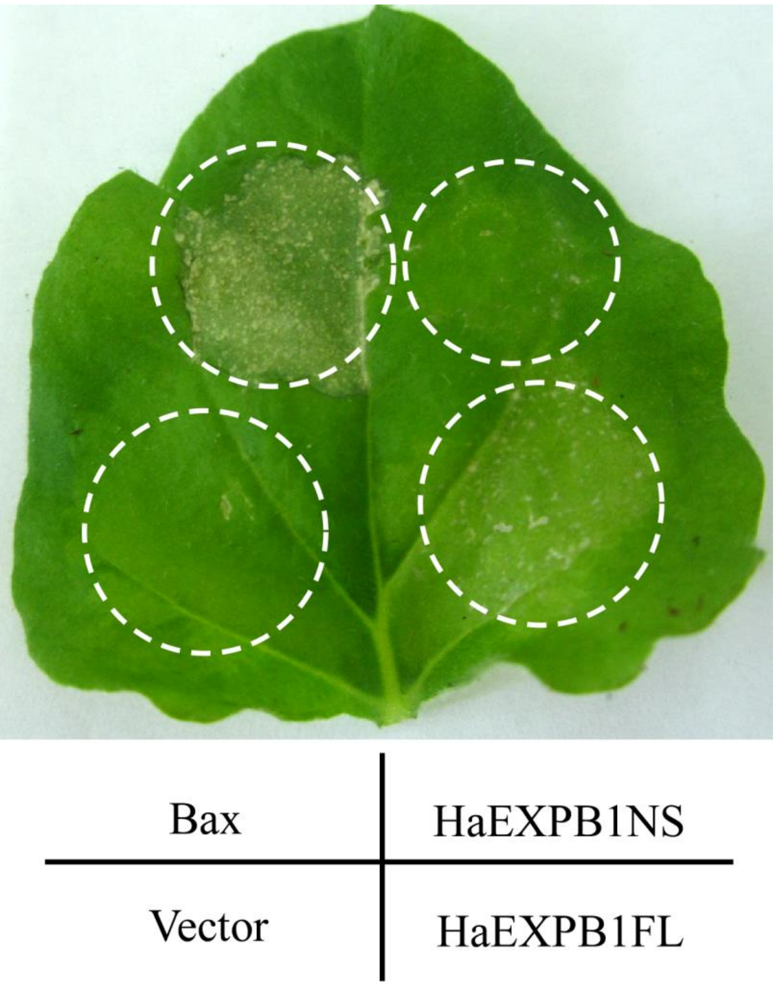

Supplemental Fig S1. Transient expression HaEXPB1 induces cell death in *N. benthamiana*. Agroinfiltration was performed on one leaf with *Agrobacterium tumefaciens* carrying an empty vector control (pYBA1143), a positive control (Bax), constructs with full-length HaEXPB1 (HaEXPB2FL), or constructs lacking a signal peptide sequence HaEXPB1(HaEXPB1NS), respectively. Cell death symptoms were assessed at 5 dpi.

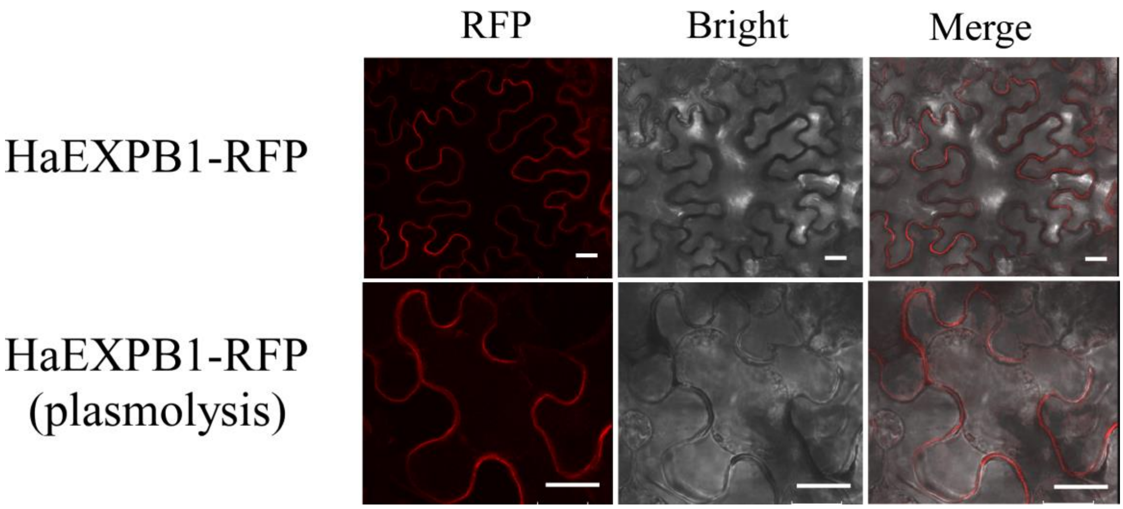

Supplementa Fig S2. Subcellular localization of transiently expressed HaEXPB1-RFP fusions in *N. benthamiana* leaves. The upper panel was HaEXPB1-RFP in normal cell, the lower panel was HaEXPB1-RFP in plasmolysed cell. Scale bar = 20 μm.

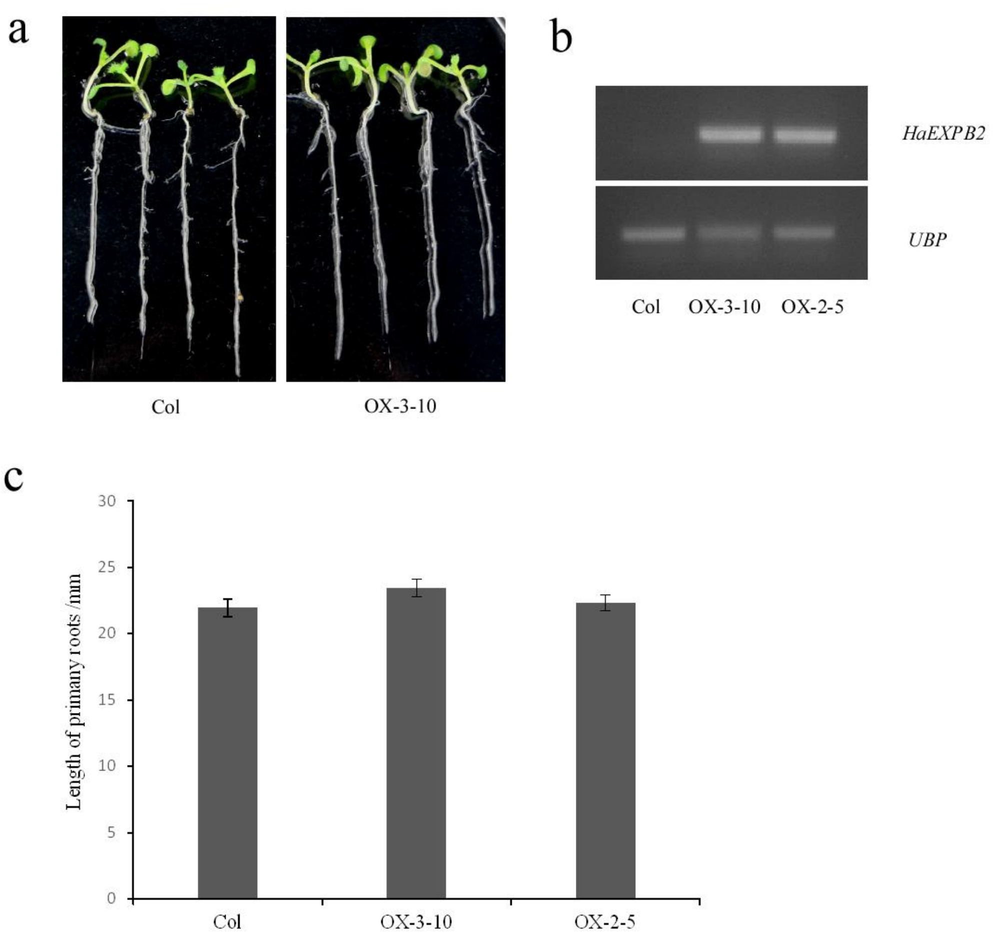

Supplemental Fig S3. Over expressed HaEXPB2 in *A. thaliana*

(a) The phenotype of over-expressing HaEXPB2 *A. thaliana*.

(b) RT-PCR confirmed the expression level of HaEXPB2 in transgenic *A. thaliana* lines OX-3-10, OX-2-5 compared with the WT (Col). (c) The root growth of HaEXPB2 *A. thaliana* lines OX-3-10, OX-2-5 and WT. Data are presented as means ± SD from 20 plants.
